# Effectiveness of Mobiderm Autofit in the Intensive Phase of Breast Cancer-Related Lymphedema Treatment: A Case Series

**DOI:** 10.1089/lrb.2022.0079

**Published:** 2023-12-22

**Authors:** Sławomir Mazur, Dorota Szczęśniak, Hanna Tchórzewska-Korba

**Affiliations:** Department of Rehabilitation, Curie National Research Institute of Oncology (MSCNRIO), Warsaw, Poland.

**Keywords:** compressive garment, complete decongestive therapy, intensive phase, self-management, breast cancer-related lymphedema

## Abstract

**Background:**

The objective of this case series was to evaluate the effectiveness of wearing Mobiderm^®^ Autofit compressive garment as part of the complete decongestive therapy (CDT) of upper limb lymphedema.

**Materials and Methods:**

Ten women and men with stage II breast cancer-related lymphedema underwent a CDT intensive phase for 12 days, combining Mobiderm Autofit compression garment with manual lymphatic drainage. Arm volume was calculated with the truncated cone formula using circumferential measurements taken at each appointment. The pressure under the garment and the overall satisfaction of patients and physicians were also assessed.

**Results:**

The mean (standard deviation [SD]) age of the patients was 60.50 (11.70) years. The mean (SD) lymphedema excess volume decrease was 343.11 (266.14) mL, which represents a 36.68% decrease between day 1 and day 12, whereas the mean (SD) absolute volume difference was 420.03 (251.27) mL corresponding to a 10.12% decrease during this same period. The mean (SD) device pressure by using the PicoPress^®^ was 30.01 (0.45) mmHg. The majority of patients were satisfied with the ease of use and the comfort of wearing Mobiderm Autofit. Such positive assessment was confirmed by the physicians. During this case series, no adverse event was reported.

**Conclusion:**

A lymphedema volume decrease of the upper limb was reported after 12 days of treatment with Mobiderm Autofit during the CDT intensive phase. Moreover, the device was well tolerated, and its use was appreciated by the patients and the physicians.

## Introduction

Breast cancer is the most frequently diagnosed cancer in the female population.

During surgery for cancer, the related lymph nodes are often removed. This disrupts the flow of lymph, which can lead to swelling, called lymphedema. Radiotherapy of the axillary lymph nodes is often combined to surgery, increasing the occurrence of lymphedema.^1^

Breast cancer-related lymphedema (BCRL) may occur immediately after surgery or radiotherapy, or months or even years later.^2^ In a meta-analysis, it was shown that approximately one in five patients who had undergone breast cancer surgery with lymph nodes removal will develop lymphedema,^3^ which has a significant negative impact on patients' quality of life after breast cancer treatment inducing high rates of depression and anxiety in addition to lymphedema-related disability. However, the lack of BCRL diagnostic criteria leads to high variability in reported incidence from less than 5% to more than 50%, largely dependent on patients' individual risk factors.^4^

The main symptom of BCRL is swelling of the arm on the side where lymph nodes were removed. Other symptoms of lymphedema may include feeling of fullness, heaviness, or tightness in the arm, as well as pain. Moreover, trouble moving the shoulder and hand joints, weakness in the arm, and abnormal sensations such as pin and needles can be observed. Concerning the skin issues, thickening or changes of the skin appearance or skin sores can also develop.

Swelling may become severe. The presence of this lymphatic fluid results in inflammation, followed by fibrosis and sclerosis, then adipose tissue differentiation, and finally progression to chronic irreversible lymphedema with fibrotic changes in the soft tissue.^5^

The International Society of Lymphology (ISL) has defined four stages of lymphedema evolution.^6^ While stage I early edema improves with limb elevation, from stage II onward, tissue swelling and pitting are evident and will require a proper course of complete decongestive therapy (CDT), the current international standard of care for managing lymphedema.^6^ Due to the multiple objectives of CDT, it includes the combination of compression bandages, elevation, specialized massage techniques, known as manual lymphatic drainage (MLD), combined with skin care and exercises.^7^

The treatment of lymphedema from stage II onward will start with the intensive phase, its primary objective being the maximum reduction of the lymphedema volume by applying sufficient pressure on the limb, depending on the localization and the stage of the lymphedema;^8^ it may last few days up to 8 weeks, according to the usual practice at the clinical center. It is then followed by the maintenance phase, which may be lifelong.^7^ Early commencement of BCRL treatment is recommended, especially at stage I and stage II, to prevent further progression to fibrous and fatty tissue.

CDT has been shown to be effective in a large number of studies demonstrating significant limb volume reductions, improvement in limb appearance, reduction in symptoms, improvement in quality of life, and diminution of infections after treatment.^5,7,9,10^

**FIG. 1.**
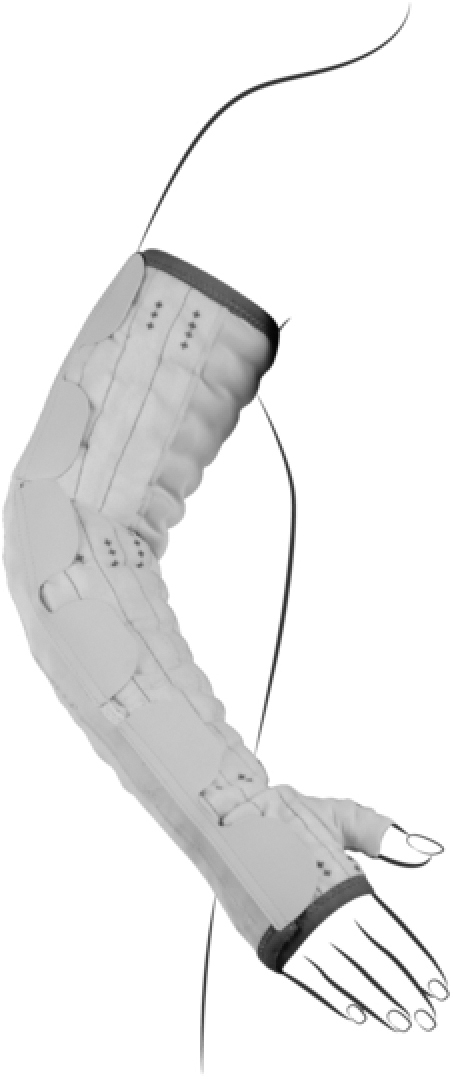
Auto-adjustable Mobiderm^®^ Autofit.

Mobiderm^®^ Autofit is a system to mobilize edematous or indurated subcutaneous tissue of the affected limb and is mainly intended for the treatment of upper or lower limb lymphedema during the maintenance phase. It is presented in the form of self-adjustable garments, including a standard sleeve with mitten and thumb, which can be adapted to the morphology of each patient and easily tightened (Fig. 1). The patient will be able to adjust the garment more or less tightly on a daily basis.

Mobiderm has garnered interest through clinical studies in the treatment of lower and upper limb lymphedema.^9–11^ This CE-marked device has a well-established use in both outpatient and inpatient settings for the maintenance phase of the treatment of lymphedema.^11^

While it has also been used in practice during the intensive phase according to physicians and physiotherapists feedback, the efficacy of Mobiderm Autofit during this phase has never been reported.

This publication aims to report the effectiveness of wearing Mobiderm Autofit during the intensive phase of BCRL treatment through a prospective case series on 10 patients.

## Materials and Methods

### Patients

Subjects presenting with secondary unilateral upper limb stage II ISL^6^ lymphedema were recruited at the Department of Rehabilitation at the Maria Sklodowska-Curie National Research Institute of Oncology (Warsaw, Poland), between May and October 2021.

The research was completed in compliance with the Declaration of Helsinki and the International Conference on Harmonization Good Clinical Practice Guidelines (ICH-GCP). Subjects were informed about the objectives, the treatment, the type of study-related procedures, the use of their collected personal data, and they signed an informed consent form to participate in this case series.

### Treatment

Mobiderm Autofit was used for 24 hours, day and night, according to the physicians' recommendations. Patients were instructed on how to adjust the device on their arm and on the position of the Velcro straps to tighten the subsequent levels along the arm (Fig. 1).

The experimental protocol followed the routine used by the hospital during the intensive phase of lymphedema treatment: it was combining compression and MLD, in accordance with the recommendations of the ISL.^6^ The duration of the treatment was 12 consecutive days. The patients were required to attend 10 visits at the outpatient clinic during the treatment period, from Monday to Friday.

### Assessments and outcome measures

Sociodemographic data (age, weight, body mass index [BMI]), clinical history (age at cancer diagnosis, type of cancer treatment, stage of the lymphedema, and duration), as well as records of concomitant medication and previous treatments related to lymphedema, such as CDT, were collected from the patient's medical file.

The limb circumference of both the affected and healthy arms was measured at day 1 and each visit day up to day 12, the final visit, as performed in routine care.^12^

The limb circumference was measured every 5 cm, above (four measurements) and below (four measurements) the fold of the elbow (start measurement point) and at the metacarpal-phalangeal joints, at the thumb base and at the wrist.

The severity of lymphedema was categorized as mild (<20% increase in extremity volume), moderate (20%–40%), or severe (>40%).^13^

Measurement of the device pressure using Picopress^®^ was performed daily from day 1 to day 12.

Satisfaction was assessed by the patient including ease of use with facility to put, to adjust, and to remove (4-point scale: very easy, easy, difficult, very difficult), comfort (4 point-scale: very comfortable, comfortable, uncomfortable, very uncomfortable), pressure of the device (4-point scale: totally bearable, quite bearable, endurable with difficulty, intolerable), and effect of Mobiderm Autofit on skin suppleness (4-point scale: not at all, lightly, moderately, totally). Satisfaction was also asked to the physicians including facility of use (yes/no) and less time consuming than using bandages (yes/no).

### Safety assessment

Reported or observed adverse events, in particular skin tolerability, were collected at each visit.

### Data analysis

The volume of the arm was calculated using the truncated cone formula: *H* × (*C*^2^ + *C* × *c* + *c*^2^)/12π, where *H* = cone height, *C* = cone top circumference, and *c* = cone base circumference.

The lymphedema excess volume was calculated by the difference between the measured volume of the affected upper limb and the normal contralateral limb; it is expressed in milliliters and in percentage.

The percentage change in volume of the lymphedematous limb (%) was calculated using the following formula: (Vi − Vf)/(Vi) × 100 (Vi: the initial volume of the lymphedematous limb; Vf: the final volume of the lymphedematous limb is also known as absolute volume difference).

Descriptive statistics was determined as mean (standard deviation [SD]) for continuous variables, median (minimum − maximum) for discrete variables, and number (*n*) and percentage (%) for categorical variables. This was performed for demographic characteristics, all clinical measures, and qualitative assessments on the overall population.

## Results

### Baseline patients and lymphedema characteristics

A total of 10 patients, 8 female and 2 male patients, with the diagnosis of BCRL were evaluated. The mean age was 60.50 (11.70) years, with a range between 46 and 81 years. The patients' mean body weight was 83.86 (10.50) kg, with a mean BMI of 31.30 (4.06); three patients (30%) presented as overweight and seven (70%) were obese (BMI ≥30).

All patients (*N* = 10) had an upper limb lymphedema secondary to breast cancer, treated with surgery, combined with radiotherapy in nine patients, chemotherapy in eight patients, and immunotherapy in one patient. The mean duration between cancer diagnosis and first treatment day was 7.90 (5.11) years.

The mean (SD) duration of lymphedema before starting the treatment was 6.1 (3.4) years (median = 5.67 years). BCRL had been diagnosed within the previous 5 years in four patients, and the other six patients had a lymphedema history more than 5 years.

**FIG. 2.**
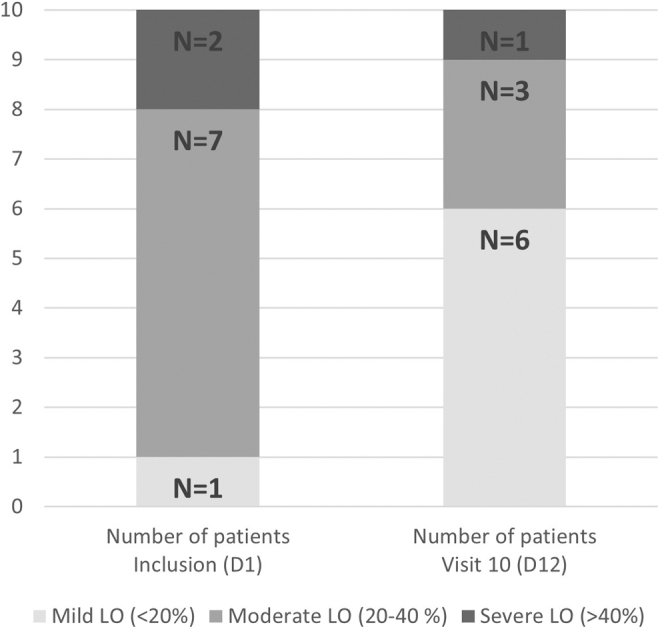
LO severity categories for the volume excess. Number of patients (*N* = 10). LO, lymphedema.

When the information on the stage of lymphedema was available (*n* = 8), one was a stage II-A lymphedema and the other seven were a late stage II-B. At day 1, one patient (10%) presented a mild lymphedema (<20% excess volume) and nine patients (90%) presented a moderate-to-severe lymphedema (>20% excess volume) (Fig. 2).

All patients had already been treated for their lymphedema before inclusion, and seven (70%) had already received a CDT, with a mean number of 3.86 (3.34) CDT courses since the diagnosis.

In six of seven patients, the device used during the last CDT was bandaging incorporating Mobiderm bandage (i.e., not autofit garment) and long stretch bandages (Biflex^®^ or another bandaging). No information was available for one patient.

### Lymphedema volume decrease

All patients wore Mobiderm Autofit over the 12 days of follow-up.

The excess limb volume at baseline was severe in two subjects (63% and 46%, respectively), moderate in seven patients (20.5%–33.3%), and mild in one patient. At the end of the treatment period, there was at least a 50% decrease from baseline of the excess volume in four patients. In five patients, the excess volume decrease was between 25% and 33%. In one subject with 2-year history, the high baseline excess limb volume did not change over the course of treatment; this patient had previously received two courses of CDT, which had not been efficient (Table 1).

**Table 1.** Lymphedema Parameters per Patient at Baseline (D1) and at the End of the Treatment Period (D12)—(10 Patients)

Subject No.	1	2	3	4	5	6	7	8	9	10
Baseline (D1)										
LO duration, years	7.4	5.9	4.8	5.4	8.2	2.2	13.8	2.0	7.3	3.8
AUL volume, mL	5061.2	3606.6	5433.8	4767.4	3202	3065.6	3800.9	3991.7	4503.4	3255.8
Volume excess, mL (%)	1964.5 (63.4)	721.9 (25.0)	851.60 (18.6)	1088.4 (29.6)	575.0 (21.9)	521.3 (20.5)	723.13 (23.5)	1255.9 (45.9)	1125.5 (33.3)	710.8 (27.9)
End treatment (D12)										
Volume excess, mL (%)	988.3 (31.5)	539.7 (19.2)	268.4 (5.9)	727.4 (20.4)	390.5 (15.1)	254.3 (9.9)	503.3 (17.2)	1233.3 (46.0)	844.6 (25.5)	357.3 (15.7)
Excess volume decrease D1–D12, %	50	25	68	33	32	51	30	1	25	50
AUL volume decrease D1–D12, %	18	7	12	10	7	9	10	2	8	19

AUL, affected upper limb; D, day; D1–D12, change between day 1 and day 12; LO, lymphedema.

Considering that earlier treatment is optimal for the best results,^6^ it is interesting to note that three of the four major improvements (reduction by more than 50%) were observed in patients with a lymphedema duration shorter than 5 years.

If we consider all patients, the mean lymphedema excess volume decrease from day 1 to day 12 was 343.11 (266.14) mL, and the mean absolute volume difference was 420.03 (251.27) mL, which represents a 36.68% and 10.12% decrease, respectively. The number of patients presenting a moderate-to-severe lymphedema decreased from nine (90%) at day 1 to four (40%) at day 12 (Fig. 2).

### Device pressure

The mean device pressure for all patients (*N* = 10) measured at all visits by using the PicoPress was 30.01 (0.45) mmHg.

### Skin suppleness

**FIG. 3.**
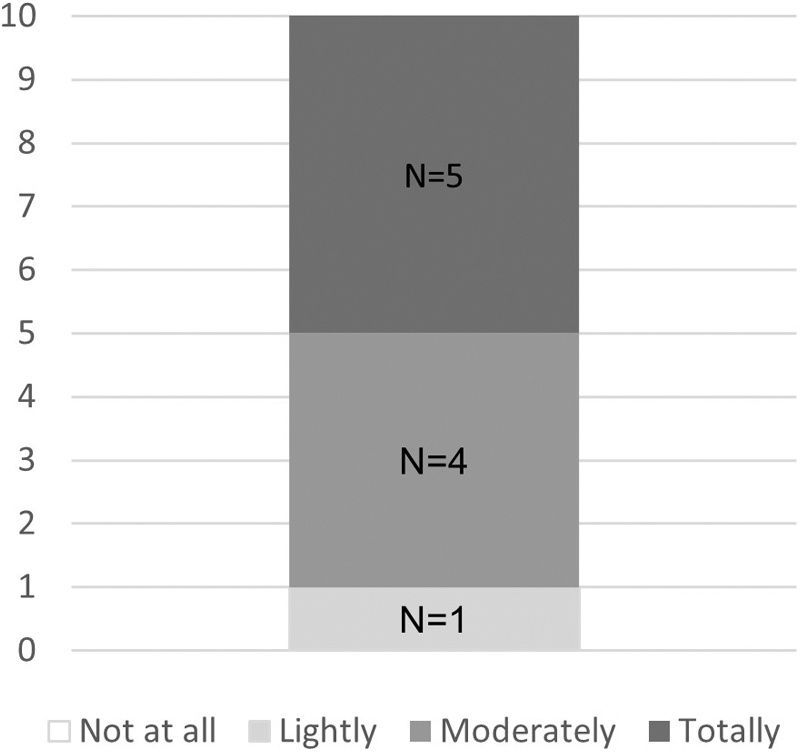
Patients' opinion on the improvement of their skin suppleness. Number of patients (*N* = 10).

All patients reported an improvement of their skin suppleness between the first and last day of decongestion phase, with nine patients (90%) presenting a total or moderate improvement (Fig. 3).

### Use and satisfaction questionnaires

After 12 days of wearing the device, all the 10 patients found the device very easy or easy to put on, 9 being able to put it on alone, 7 (70%) found it very easy or easy to adjust, and all the 10 patients found it very easy or easy to remove. Patient satisfaction was high with all patients rating the device as comfortable to very comfortable to wear (*N* = 10). Moreover, nine patients (90%) found the device pressure totally or quite bearable.

Regarding the physician's opinion, they were very satisfied or satisfied regarding the device application for all patients, as it was assessed as easy to use and fast to put on.

### Safety results

During the 12-day follow-up, no patient reported any adverse event. The Mobiderm Autofit device was well tolerated by the patients during the intensive phase of lymphedema treatment.

## Discussion

This is the first case series assessing the effectiveness of wearing Mobiderm Autofit in addition to MLD in the intensive phase of CDT, per the recommendations of the ISL.^6^ Ten patients with upper limb lymphedema secondary to breast cancer surgery and radiotherapy were followed. After treatment, the mean decrease of excess limb volume due to lymphedema between day 1 and day 12 was 36.68%.

These results are comparable to those reported in a cohort study by Vignes et al. in which 357 women with secondary arm lymphedema were treated with bandaging, lymphatic drainage, exercise, and skin care. At the end of the intensive decongestive phase (mean ± SD: 11.8 ± 3.3 days), the mean (SD) relative excess volume reduction was 36% (14).^14^

Moreover, in the current case series, the percentage reduction of the absolute volume difference was 10.3% between day 1 and day 12. These results are comparable to those reported in a clinical study by Ozcan et al., wherein 37 women with BCRL were treated for 3 weeks with compression bandaging combined with MLD and the recommended standard of care. The reduction of the volume of the affected limb corresponded to 8.3% decrease.^5^

The use of Mobiderm Autofit during the intensive phase induced a clinically relevant lymphedema excess volume decrease (at least 25% decrease) in nine patients despite the fact that these patients had a rather long history of lymphedema between 2 and 13.8 years (median = 5.67 years) and all had received at least one course of CDT in the past. Therefore, these patients could be considered more difficult to treat than *de novo* patients, which make these results clinically interesting to further investigate. The clinical relevance of these results for the patients was reported in their good satisfactory assessments.

It is important to note that the compression class level (CCL) is of central importance, to qualify the compression garments. For arm and hand lymphedema, they are usually of CCL 2 (23–32 mmHg).^8^ In addition, the literature has shown that a low pressure may be better tolerated and can give similar results to high pressure bandaging.^15,16^ In addition, to be optimal, the pressure of compression bandaging must always be individually adapted to the patient's limb circumference, tissue consistency, and mobility. The pressure applied with Mobiderm Autofit was stable, in average 30 mmHg, across the treatment period.

Mobiderm is available as different presentations such as paddings, bandages, tailored garments, or standard garments such as Mobiderm Autofit. The results reported in this case series are consistent with those of published clinical studies reporting the efficiency of Mobiderm bandages during the intensive phase.

The POLIT study was performed in 306 females with unilateral upper or lower limb lymphedema treated with daily multilayer bandaging during the CDT intensive phase. In the group treated with Mobiderm padding, the median reduction in excess volume was 27.4% after the first 5 days of CDT compared with 22.2% with the other padding types.^9^ Furthermore, in the MOBILITY study, 50 female patients with BCRL were randomized to receive either conventional multilayer bandaging or mobilizing bandaging using Mobiderm.^10^ The reduction of excess limb volume in the Mobiderm group was higher (57.3%) than that in the control group (25.1%) after 15 days of treatment. These results showed the benefit of using the Mobiderm multilayer compression bandaging for lymphedema reduction in the intensive phase. This higher efficacy than other types of bandages could be attributed to the foam squares that compose the Mobiderm device.

Moreover, in the current case series, the physicians reported that, in comparison to multilayer bandaging previously used, Mobiderm Autofit was more comfortable according to patient satisfaction questionnaire, easier to use, and faster to put on. These properties allowed nine of the 10 patients to be able to put on the device independently. Of note, the application of bandaging requires more time and skills to properly apply them on the patient's limb with a sufficient pressure and without any injury. In addition, such bandaging application requires an external person, leading the patient to depend on a third party.

Thus, the advantages of the Mobiderm Autofit for the patients are the ease and the speed of application, the stable effect since it reproduces the necessary pressure every day without risk of injury, and is comfortable to wear both day and night. Considering the positive properties of these adjustable sleeves, it could be anticipated that, from the first days of the important intensive CDT phase, the patient could self-manage the application of Mobiderm Autofit at home, therefore increasing his level of autonomy. Only few visits at the clinical center would still be necessary to check the progression of the lymphedema volume and potentially adjust the device, in addition to limited self-lymphatic drainage or lymphatic drainage that could be performed by a private physiotherapist. The impact of reducing the number of decongestive lymphatic therapy must be evaluated in a future clinical study. These conditions of use would reduce the costs of the treatment, which would add a general benefit for patients and/or insurance companies.

It should be noted that nine patients reported an increase in the skin suppleness after the 12-day treatment period. The Mobiderm technology is supposed to facilitate the flow of lymphatic fluid and optimize the efficiency of the drainage; therefore, the frequent indurated zones would be softened.

The absence of any reported adverse event is important for these patients with fragile cutaneous tissues. This confirms the very good profile already known with Mobiderm Autofit, with sparse local adverse events recorded in the materiovigilance database (data on file).

The findings reported here are based on a small number of patients who went through a course of CDT intensive phase with Mobiderm Autofit. There was no control and confounding factors, and uncontrolled bias may have occurred, well-known limitations of such case series. While the trends observed provide interesting insight in the potential benefit of Mobiderm Autofit, an additional trial on a larger population and using a comparative design is needed to verify whether the findings from this case series would generalize to a broader population.

## Conclusion

The case series showed that most patients wearing Mobiderm Autofit during the CDT intensive phase reported a clinically relevant decrease in their lymphedema volume after 12 days of treatment. Pressure measurements showed that it was possible to reach a stable 30 mmHg pressure level under Mobiderm Autofit. Moreover, the device was well tolerated and appreciated by patients and physicians for its ease of use by the patients themselves.
